# Air pollutants and hospitalization due to pneumonia among children. An ecological time series study: Poluentes atmosféricos e internações por pneumonia em crianças. Estudo ecológico de série temporal

**DOI:** 10.1590/1516-3180.2014.00122601

**Published:** 2015-08-21

**Authors:** Tassia Soldi Tuan, Taís Siqueira Venâncio, Luiz Fernando Costa Nascimento

**Affiliations:** I Medical Student. Department of Medicine, Universidade de Taubaté (Unitau), Taubaté, São Paulo, Brazil.; II MD, PhD. Assistant Professor, Department of Medicine, Universidade de Taubaté (Unitau), Taubaté, São Paulo, Brazil.

**Keywords:** Air pollutants, Pneumonia, Child health, Ozone, Carbon monoxide., Poluentes do ar, Pneumonia, Saúde da criança, Ozônio, Monóxido de carbono.

## Abstract

**CONTEXT AND OBJECTIVE:**

Exposure to air pollutants is one of the factors responsible for hospitalizations due to pneumonia among children. This has considerable financial cost, along with social cost. A study to identify the role of this exposure in relation to hospital admissions due to pneumonia among children up to 10 years of age was conducted.

**DESIGN AND SETTING:**

Ecological time series study using data from São José dos Campos, Brazil.

**METHODS:**

Daily data on hospitalizations due to pneumonia and on the pollutants CO, O_3_, PM10 and SO_2_, temperature and humidity in São José dos Campos, in 2012, were analyzed. A generalized additive model of Poisson's regression was used. Relative risks for hospitalizations due to pneumonia, according to lags of 0-5 days, were estimated. The population-attributable fraction, number of avoidable hospitalizations and cost savings from avoidable hospitalizations were calculated.

**RESULTS:**

There were 539 admissions. Exposure to CO and O_3_ was seen to be associated with hospitalizations, with risks of 1.10 and 1.15 on the third day after exposure to increased CO concentration of 200 ppb and ozone concentration of 20 µg/m^3^. Exposure to the pollutants of particulate matter and sulfur dioxide were not shown to be associated with hospitalizations. Decreases in CO and ozone concentrations could lead to 49 fewer hospitalizations and cost reductions of R$ 39,000.00.

**CONCLUSION:**

Exposure to certain air pollutants produces harmful effects on children's health, even in a medium-sized city. Public policies to reduce emissions of these pollutants need to be implemented.

## RESUMO

**CONTEXTO E OBJETIVOS:**

A exposição a poluentes do ar é um dos fatores responsáveis pelas internações por pneumonias em crianças. Esse desfecho tem custo financeiro considerável, além do custo social. Estudo para identificar o papel dessa exposição nas internações em crianças com até 10 anos de idade foi desenvolvido.

**TIPO DE ESTUDO E LOCAL:**

Estudo ecológico de séries temporais com dados de São José dos Campos, Brasil.

**MÉTODOS:**

Dados diários de internações por pneumonia, dos poluentes CO, O_3, _PM10, SO_2_ além de temperatura e umidade de São José dos Campos, em 2012, foram analisados. Utilizou-se modelo aditivo generalizado da regressão de Poisson e foram estimados os riscos relativos para internações por pneumonia segundo defasagens de 0 a 5 dias. Foram calculadas a fração atribuível populacional, as internações evitáveis e a economia nos custos das internações evitáveis.

**RESULTADOS:**

Foram 539 internações. Exposição ao CO e O_3_ se mostraram associadas às internações, com riscos de 1,10 e 1,15 no terceiro dia após a exposição decorrentes de aumento nas concentrações do CO em 200 ppb e nas concentrações de ozônio em 20 µg/m^3^. Exposições aos poluentes material particulado e dióxido de enxofre não se mostraram associados às internações. Diminuição nas concentrações de CO e O_3_ poderiam reduzir em 49 internações e de R$ 39 mil nos custos.

**CONCLUSÃO:**

Mesmo em uma cidade de médio porte, exposição a determinados poluentes do ar causa efeito danoso à saúde da criança, sendo necessária a implantação de políticas públicas para redução da emissão desses poluentes.

## INTRODUCTION

Among children, pneumonia is a multifactorial disease for which the risk factors include poor nutritional status, lack of breastfeeding, low parental education, low birth weight, low maternal age, low weight gain during pregnancy, presence of smokers in the environment, parity and overcrowding in the home.^1^

In Brazil, in 2012, there were approximately 300,000 hospitalizations due to respiratory diseases, of which around 50,000 were in the state of São Paulo. Respiratory diseases also generate expense for the National Health System, considering that in 2012, in Brazil, spending on hospital admissions due to pneumonia in this age group was close to R$ 200 million (1 US $ ≈ R$ 2.20), of which R$ 40 million related to the state of São Paulo. Data from 2010 showed that, of the 55,000 deaths due to pneumonia, 18,000 were in the state of São Paulo, and 2% were among children up to 10 years of age.^2^ Exposure to air pollutants was highlighted, regardless of which of the factors mentioned above were present.

Air pollutants can be primary, i.e. those emitted directly by emission sources, or secondary, like ozone, which are formed through chemical processes between primary pollutants and natural compounds in the atmosphere. They consist of a heterogeneous mixture of substances, including carbon monoxide (CO), ozone (O_3_), sulfur oxides (SOx), nitrogen oxides (NOx), organic compounds (hydrocarbons, alcohols, aldehydes, ketones, organic acids and halogen compounds) and particulate matter (PM). PM composition depends on the pollution source and on environmental factors such as meteorological conditions, industrial activity and vehicle traffic density. The inhalable particles may be fine (PM2.5; < 2.5 µm), which may reach the pulmonary alveoli, or coarse (PM10; 2.5-10 µm), which are retained in the upper airways.^3^ ^4^ ^5^ ^6^ ^7^ ^8^ ^9^ ^10^ ^and^ 11

Studies conducted in major centers such as São Paulo,^6^ Rome,^12^ Taipei^13^ and Rio de Janeiro^8^ and in some medium-sized Brazilian cities, such as São José Campos,^3^ ^5^ Piracicaba^14^ and Cubatão,^7^ have identified an association between hospital admissions due to respiratory diseases and exposure to air pollutants, such as coarse and fine particulate matter (PM10 and PM2.5), ozone (O_3_), sulfur dioxide (SO_2_) and carbon monoxide (CO).

## OBJECTIVE 

This study aimed to estimate the association between exposure to air pollutants and hospital admissions due to pneumonia among children up to 10 years of age in the city of São José dos Campos, state of São Paulo.

## METHODS

This was an ecological time series study using data on hospitalizations due to pneumonia (International Classification of Diseases, ICD, 10^th^ revision, sections J12.0 to J18.9) in the public health system (six hospitals), from children of both sexes up to 10 years of age, living in São José dos Campos. The study was carried out between January 1, 2012, and December 31, 2012. Hospital admission data were obtained from the DATASUS system.^2^

The environmental agency of the state of São Paulo (CETESB),^15^ a government department that has a measuring station in São José dos Campos, provided the concentration values for particulate matter (PM10), sulfur dioxide (SO_2_) and ozone (O_3_), quantified in µg/m^3^, and carbon monoxide (CO) measured in ppb, along with minimum temperature and relative air humidity data. PM10, SO_2_ and CO were analyzed using mean daily values and O_3_ was analyzed using eight-hour values, and the maximum value was applied.

São José dos Campos is a city in southeastern Brazil located between the cities of São Paulo and Rio de Janeiro, in the middle region of the Paraíba valley, 84 km east of the state capital, and has a population of about 650,000 inhabitants. Its geographical location is at latitude 23°11' S and longitude 45°53' W. The city has a vehicle fleet of approximately 360,000 and is crossed by the Dutra highway, the country's main highway, with a flow of about 130,000 vehicles per day, among which heavy vehicles (buses and trucks) form the majority. It is an important economic center with companies focusing on technology, education and research.

Poisson's regression was used to estimate relative risks of exposure to pollutants for the outcome of hospitalization because this is an event count. A database containing daily hospital admission data together with all the pollution and climatic variables was constructed. This took into account lags from zero to five days, because the effects of exposure to pollutants can be seen not only on the same day, but also on later days. Moreover, there is no consensus regarding the size of this window. Therefore, a generalized additive Poisson's regression (GAM) model was chosen. Models containing the four pollutants simultaneously and adjusted for minimum temperature, relative humidity, seasonality and day of the week were analyzed.

The results were expressed as relative risk values. The increase in relative risk (RR-INC) was calculated using the formula RR-INC (%) = (exp^(coef * INC)^ - 1) * 100, where increased concentrations (INC) of CO at 200 ppb, 10 µg/m^3^ for PM10, 5 µg/m^3^ for SO_2_ and 20 µg/m^3^ for O_3_ were assigned in the eventuality of a significant association between exposure to a particular pollutant and hospitalization. The resultant population-attributable fraction provided numbers of hospitalizations attributed to increases in pollutant concentrations. The financial costs that would be avoided through reductions in hospital admissions were calculated using the mean hospital costs provided by the DATASUS website.

## RESULTS

Five hundred and thirty-nine children were hospitalized during the study period. This comprised a mean of 1.47 hospitalizations per day (standard deviation, SD = 1.57), with a minimum of zero and maximum of 11 admissions. The mean concentrations of the pollutants (µg/m^3)^, standard deviations and minimum and maximum are shown in Table 1.

**Table 1:**
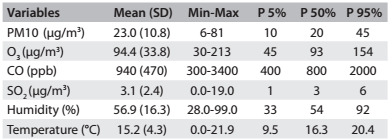
Descriptive analysis showing means, standard deviations (SD), minimum and maximum values (Min-Max) and 5%, 50% and 95% percentiles (P 5%, P 50% and P 95%) of the study variables. São José dos Campos (SP), Brazil, 2012

The behavior of ozone over the period did not show any clear seasonality. The 140 µg/m^3^ standard value was exceeded on four days and the air quality was considered to be only moderate on another four days. CO, PM10 and SO_2_ demonstrated seasonal patterns with peak concentrations in the cold months (data not shown).

Table 2 presents the correlation matrix for the study variables (environmental pollutants, climate variables and number of hospitalizations). We observed significant correlations between the pollutants, except between CO and O_3_.

**Table 2:**
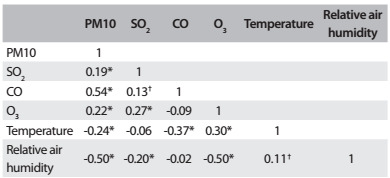
Pearson´s correlation matrix between atmospheric variables. São José dos Campos (SP), Brazil, 2012

*P-value < 0.01; ^†^P-value < 0.05

Table 3 presents Poisson's regression coefficients and standard errors for the pollutants studied on all the lag days analyzed (up to five days after the exposure). The effects on hospitalizations were not immediate: they were observed on lag day 3 and lag day 4 for CO and between lag days 2 and 5 for O_3_.

**Table 3:**
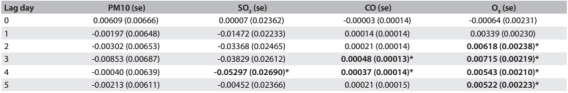
Regression coeffients and standard errors (se) for the pollutants of particulate matter (PM10), sulfur dioxide (SO_2_), carbon monoxide (CO) and ozone (O_3_) on all the lag days analyzed. São José dos Campos (SP), Brazil, 2012

*P-value < 0.05; se = standard error

**Figure 1:**
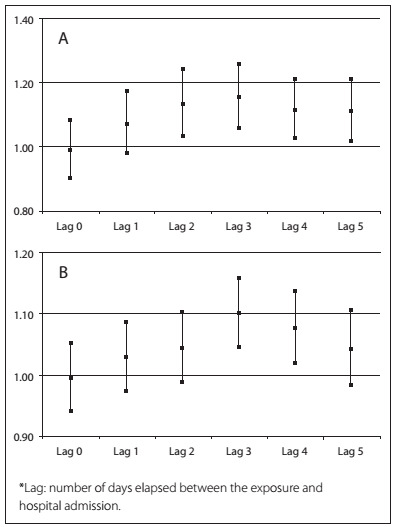
Relative risks and 95% confience intervals for increases in (A) ozone concentration (20 μg/m^3)^ and (B) carbon monoxide concentration (200 ppb), on lag days* 0 to 5. São José dos Campos (SP), Brazil, 2012.

The relative risks and 95% confidence intervals (CI) were calculated from the values supplied by the generalized additive model. The relative risks for CO were RR = 1.08 (95% CI: 1.02 to 1.14) for lag day 3 and RR = 1.10 (95% CI: 1.04 to 1.16) for lag day 4; and for ozone, RR = 1.005 (CI: 1.003 to 1.007) on lag day 2, RR = 1.007 (CI: 1.005 to 1.009) on lag day 3 and RR = 1.004 (CI: 1.002 to 1.006) on lag day 4. Figure 1 (a) and (b) show the relative risks regarding ozone and carbon monoxide, for increases of 20 µg/m^3^ in ozone concentration and 200 ppb in carbon monoxide concentration. A decrease of 20 μg/m^3^ in ozone concentration implies a decrease of up to 15 percentage points (95% CI: 5.5 to 24.6) in the relative risk of hospitalization due to pneumonia. A decrease of 200 ppb in the CO concentration implies a decrease of up to 10 percentage points. The number of hospital admissions would reduce by 49 patients, with a reduction in costs of around R$ 39,000.00, given that the average cost of hospitalization is R$ 800.00.

## DISCUSSION

This study identified the importance of exposure to CO and O_3_ in the genesis of hospitalizations due to pneumonia among children in São José dos Campos, state of São Paulo. The effects were evident on the third and fourth days after exposure to CO and on the second to fifth days after exposure to ozone.

This study identified a lower number of hospitalizations (539 reports) than a previous study on other pollutants,^3^ which identified 1300 cases in the same city, using data from 2000 and 2001. The present study also identified decreases of around 50% in PM10 and SO_2_ concentrations, compared with the previous study.^3^

These findings relating to CO in the city of São José dos Campos coincide with the results from a study conducted in São Paulo by Gouveia et al.^6^ among patients aged up to five years, even though the age group analyzed in our study was up to ten years. Their mean CO concentration was 3.24 ppm in São Paulo, i.e. 3.5 times higher than that found in our study (0.94 ppm), thus indicating that even at concentrations 70% lower, the harmful effects of exposure to CO are still present.

Similarly, exposure to mean CO concentrations of the order of 1.26 ppm showed an association with increased chance of hospitalization due to pneumonia (up to 27% higher) in a study carried out in Taiwan.^13^ In that study, despite the lack of a biologically plausible explanation for the association between CO exposure and admissions due to pneumonia, the authors believed that this might be explained by the fact that CO is a good indicator of air pollution (incomplete combustion), particularly associated with pollution due to vehicle emissions.^13^

Our findings also agree with the results of Fusco et al.,^12^ in which CO was the only pollutant associated with all the types of respiratory diseases studied, such as asthma, chronic obstructive pulmonary disease (COPD) and pneumonia.

Exposure to ozone was identified as a risk factor in 2012, unlike in the data from 2000-2001. Thus, there is a possible dose-response effect, since the mean ozone concentration in 2000-2001 was approximately 65 μg/m^3^ and, in 2012, it was 94.4 µg/m^3^, i.e. 50% higher. On the other hand, our results coincide with those found in Cubatão, state of São Paulo, where the effect occurred on the same day as the exposure, with similar ozone concentrations.^7^ In a study conducted in São Paulo in which the ozone concentrations were found to be nearly 70 μg/m^3^, Gouveia et al.^6^ also observed that ozone exposure played a role in hospitalizations due to respiratory diseases among children, relating both to pneumonia and to asthma. Fusco et al. also found that ozone exposure played a role in hospital admissions due to total respiratory disease and due to pneumonia among children aged 0-14 years. On lag day 1, the risk was between 5% and 8% higher, given that there was an increase in ozone concentration of 23 µg/m^3^.^12^ Chiu et al. found that ozone exposure played a role in hospital admissions due to pneumonia in Taipei, both on warm and on cold days.^13^

This increase in ozone concentrations may be associated with possible increased concentrations of NOx released by fuel-burning, mainly due to the vehicular fleet. In this, NOx is transformed into NO_2_. The vehicular fleet increased from around 180,000 vehicles in 2000 (135,000 cars) to around 330,000 in 2011 (232,000 cars) (http://www.denatran.gov.br/frota.htm), which may explain the increase in NOx concentrations with a consequent increase in ozone concentrations. This pollutant (NO_2_) was not analyzed in the present study because data on this in São José dos Campos were not yet available from CETESB. On the other hand, data were available from Sorocaba, state of São Paulo, another medium-sized city like São José dos Campos, where NO_2_ emissions were correlated with hospitalizations due to pneumonia.^16^

This study did not identify any association between exposure to PM10 and admissions. However, in a study carried out 10 years ago, exposure to this pollutant was important,^3^ since it showed associations three and four day after exposure and hospitalization. In the study of 10 years ago, the mean concentration was approximately 40 μg/m^3^, i.e. almost double the level of 23 μg/m^3^ found in 2012. The reduction in the mean concentration of PM10 over this 12-year period may have been due to policies implemented for reducing pollution, through using more suitable catalysts in cars so as to decrease exhaust emissions, renewal of the vehicular fleet, installation of filters in factory chimneys and strict monitoring of emissions of pollutants. This reduction in PM10 concentration may have resulted in a reduced number of hospitalizations due to pneumonia, thereby suggesting that there has been dose-response effect.

In a study carried out in Cubatão, state of São Paulo,^7^ PM10 exposure was correlated with subsequent hospitalization due to pneumonia. The mean PM10 concentration in Cubatão was 63.8 μg/m^3^, i.e. three times higher than the PM10 concentration in our study. In another study, on children in the city of Rio de Janeiro, with mean PM10 concentration of nearly 85 μg/m^3^, the mean peak expiratory flow was impaired.^8^ Our findings also do not agree with those of the study by Samoli et al.,^9^ in which an association with exposure to PM10 was found, with an average concentration of 43 μg/m^3^, but the outcome was hospitalization due to asthma. The fine fraction (PM2.5), which accounted for nearly 60% of the quantity of PM10, was correlated with hospitalizations due to bronchiolitis in a study carried in California.^11^A study conducted in Central Europe, which found much higher concentrations than those recommended by the World Health Organization (WHO), reaching up to 400 μg/m^3^, showed that the respiratory health of children living in the urban area of Tirana was impaired due to asthma and pneumonia.^17^

Our results reinforce the results found in Piracicaba, state of São Paulo, in which it was observed that straw-burning increases the risk of hospitalization among children due to respiratory problems, by 25%, especially during the dry months of the year, in which three times as much particulate matter (PM10) as in the other months is generated.^18^

There was also no association between exposure to sulfur dioxide and hospitalizations due to pneumonia, possibly because diesel with lower sulfur content has come into use. The sulfur content has decreased from 500 ppm to 50 and 10 ppm in the new diesel formulations S-50 and S-10, respectively. The decrease in the concentrations was from 6.2 µg/m^3^ (in 2000-2001) to 3.1 µg/m^3^ (in 2012).

Importantly, the present study used records of hospitalizations within the public health system. Thus, the results are limited to the portion of the population that uses this service. However, this portion has come to represent about 90% of all hospital admissions.^19^ On the other hand, the present results do not represent admissions through health plans or health insurance, or cases of pneumonia that were treated as outpatients.

Moreover, although the data provided by DATASUS are purely financial and not strictly appropriate for epidemiological studies, they have nevertheless been widely used in ecological studies.^20^

It is also worth noting that since this was an ecological study that did not have individual information on exposure and disease, we cannot say whether a given hospitalized individual was exposed to the pollutant. Moreover, comorbidities were not assessed.

The frequencies of certain complaints may have been underestimated and errors in coding the diagnoses may have occurred, given the technological profile of the service network. Nowadays, diagnoses are confirmed later on during hospitalization, with the aim of reducing the diagnostic errors at the time of hospital admission. It is also noteworthy that the daily measurements of exposure were considered to be homogeneous throughout the city and that individuals were similarly exposed.

In the case of ozone, the magnitude of the RR was small, but the impact of air pollution on the population's health is believed to be substantial, taking into account the considerable number of individuals exposed. Estimation of the risk to the population's health regarding air pollution is the first step in planning and implementing actions aimed towards achieving a healthier environment. Production of a technical database is critical to formulation of public policies and decisions that promote socioeconomic development and also take environmental and quality-of-life issues into account.^6^

## CONCLUSION

The data presented in this study showed that pollutants such as carbon monoxide and ozone have a role in the genesis of hospital admissions due to pneumonia among children. Therefore, implementation of policies for improving public transportation might significantly reduce the risk of hospitalizations due to pneumonia among children.
